# An isothermal amplification-based point-of-care diagnostic platform for the detection of *Mycobacterium tuberculosis*: A proof-of-concept study

**DOI:** 10.1016/j.crbiot.2021.05.004

**Published:** 2021-05-21

**Authors:** Rathina Kumar Shanmugakani, Wesley Bonam, David Erickson, Saurabh Mehta

**Affiliations:** a Institute for Nutritional Sciences, Global Health, and Technology, Cornell University, Ithaca, NY, USA; b Division of Nutritional Sciences, Cornell University, Ithaca, NY, USA; c Sibley School of Mechanical and Aerospace Engineering, Cornell University, Ithaca, NY, USA; d Arogyavaram Medical Centre, Andhra Pradesh, India

**Keywords:** Tuberculosis, *IS6110*, Helicase-dependent amplification, Point-of-care

## Abstract

The timely diagnosis of active tuberculosis disease (TB) is crucial to interrupt the transmission and combat the spread of *Mycobacterium tuberculosis* (Mtb), the causative agent for TB. Here, we demonstrate the development of a specimen-direct rapid diagnostic method for TB which consists of an isothermal amplification device, Tiny Isothermal Nucleic acid quantification sYstem (TINY), coupled with helicase-dependent amplification (HDA). HDA, an isothermal amplification technique is established over TINY using pUCIDT-AMP vector carrying *IS6110*, the target DNA sequence for Mtb. The limit of detection of this technique for detecting the *IS6110* within a threshold time of 50 min is 2.5 × 10^5^ copies of *IS6110*. HDA in TINY for TB detection was evaluated using three *IS6110*-positive Mtb strains – H37Rv, CDC 1551, and Erdman wild-type and one *IS6110*-negative *Mycobacterium avium*. For spiked oral swabs, HDA in TINY detects *IS6110* without any non-specificity in relatively short turnaround time (<1.5 h), highlighting its potential utility as a specimen-direct point-of-care diagnostic for TB. TINY does not require an uninterrupted power supply and its lightweight and small footprint offers portability and easier operation in clinical settings with poor infrastructure. Overall, HDA in TINY could serve as an efficient rapid, and portable platform for the qualitative detection of TB at the point-of-care.

## 1. Introduction

Active Tuberculosis Disease (TB) is a major health problem globally and continues to exact a high toll of mortality (Khan et al., 2019). The World Health Organization (WHO) estimated that about 10 million people were afflicted with TB in 2018 (WHO, 2020). Accurate and timely diagnosis of TB is essential to initiate treatment and implement infection control measures. However, not all the TB cases are successfully identified, and about 40% of TB cases are estimated to be undiag-nosed (Lawn, 2015; Walzl et al., 2018). These critical shortcomings of TB diagnosis impede the global efforts to combat TB imposing a large human and economic burden (Laxminarayan et al., 2009; Xin et al., 2019).

Smear sputum microscopy is the most commonly used diagnostic method for TB in clinical laboratories. Although it is very simple and easy to perform at a very low cost, it can have high inter-operator variability and consequently low sensitivity (Kik et al., 2014; Ngabonziza et al., 2016). Furthermore, it is difficult to use for TB diagnosis in children and patients who have difficulty in producing enough sputum. Moreover, sputum poses a high risk to the health care workers who collect the specimens.

After the introduction of polymerase chain reaction (PCR), several amplification-based molecular diagnostics were developed and evaluated for the diagnosis of TB (Lanzas et al., 2016; Nguyen et al., 2018; Rakotosamimanana et al., 2019; Singpanomchai et al., 2019). The WHO recommended Xpert® MTB/RIF (Cepheid, Inc., USA), a PCR-based real-time detection system for TB showed better sensitivity and specificity than smear sputum microscopy along with a turnaround time of ~2 h (Rasheed et al., 2019; Steingart et al., 2014). The Xpert® MTB/RIF however is relatively expensive, requires infrastructure, uninterrupted power supply, and maintenance which altogether limits its point-of-care (POC) applicability (Hsiang et al., 2016; Puri et al., 2016; Singpanomchai et al., 2019; Vassall et al., 2017) in many high-burden settings.

Different isothermal amplification techniques such as loop-mediated isothermal amplification, nucleic acid sequence-based amplification, and recombinase polymerase amplification are also employed in TB diagnostics (Bicmen et al., 2011; Sharma et al., 2019; Singpanomchai et al., 2019). Compared to conventional PCR, isothermal amplification techniques can provide the results rapidly without the requirement of a thermal cycler (Deng and Gao, 2015; McNerney and Daley, 2011). However, several limitations such as specificity, portability, the requirement of uninterrupted power supply, automated or semi-automated result interpretation preclude their applicability at the POC, especially in LMICs. Therefore, a TB detection system that possesses all the characteristics of an efficient diagnostic such as high sensitivity and specificity, speed, clinical specimen compatibility, inexpensive, simplicity, and no need for continuous maintenance and expertise to operate is needed for POC use. Compatibility with non-sputum samples is also desirable.

In this study, we describe a rapid and portable isothermal amplification-based platform for the qualitative POC diagnosis of TB. We exploit the helicase-dependent amplification (HDA), an isothermal amplification technique in which the helicase enzyme unwinds the double-stranded DNA eliminating the heat denaturation and thermal cycling steps as required in conventional PCR (Vincent et al., 2004). Compared to other isothermal amplification techniques, which use more than two primers or require the formation of complex DNA structures, HDA requires only two primers for amplification and forms only one double-stranded amplicon. This yields a more specific amplification while also facilitates the interpretation of the results. The utility of HDA for TB diagnosis has been reported in other studies (Barreda-Garcia et al., 2015; Barreda-Garcia et al., 2016; Shetty et al., 2017; Torres-Chavolla and Alocilja, 2011), in which the result interpretation was performed with agarose gel electrophoresis, electrochemical detection, or genomagnetic assays. However, the requirement of uninterrupted power supply, expertise, and equipment for result interpretation preclude the application of these techniques at the POC. Recently, we reported the development of Tiny Isothermal Nucleic acid quantification sYstem (TINY), an isothermal amplification device that could be used as a POC diagnostic in austere settings (Snodgrass et al., 2018). TINY consists of a temperature-regulation unit with a measurement unit placed at its center. Most of the temperature-regulation and measurement units are made with aluminium which offers a lightweight to the equipment. TINY has been designed to run the amplification reaction simultaneously for six samples. TINY can be run with a battery which can power it for 24 h. Furthermore, TINY can store the excess solar energy collected from sunlight as latent heat which can be used to run the equipment at times of interrupted power supply or no sunlight (Snodgrass et al., 2018). The low weight of 1.1 kg and its operation over battery or solar power offers POC utility. Furthermore, the results can be interpreted instantly, and the data can be transferred to the laboratory information system easily. Here, we report the development and evaluation of HDA over the TINY as a POC diagnostic platform for the qualitative detection of TB.

## 2. Materials and methods

### 2.1. Reaction conditions for HDA for the detection of Mtb

The construction of optimal HDA reaction condition commences with the primer designing for *IS6110*, an insertion sequence present in multiple copies exclusively within the Mtb complex genome (Millan-Lou et al., 2013). Different primer sets were designed using Primer3web version 4.1.0 (http://primer3.ut.ee/) according to the HDA reaction specifications such as primer length, product length, GC%, and melting temperature described in the IsoAmp® III Universal tHDA kit (Quidel, USA). The pUCIDT-AMP vector carrying the *IS6110* sequence and the designed primer sets were purchased from Integrated DNA Technologies. Inc., USA. For the establishment of optimal amplification conditions such as primer, amplification time, temperature, and concentration of reaction ingredients, HDA was performed using IsoAmp® III Universal tHDA kit in the ViiA7 real-time PCR system (Applied Biosystems, USA). A total of 10 primer sets including the one previously reported (Barreda-Garcia et al., 2016) were examined (Supplementary Table S1) for their specific amplification and threshold time. Based on the specific amplification and a shorter threshold time, the IS6110–10F (5′-caacaagaaggcgtactcgacctga-3_′_) and IS6110–10R (5′-ctcgctgaaccggatcgatgtgtact-3′) primer set that amplifies an 84 bp amplicon was chosen finally for the detection of *IS6110* by HDA (Barreda-Garcia et al., 2016). Further, the previous studies for this primer set against the isolated genomic DNA from non-Mtb strains showed that it is highly specific for *IS6110* in Mtb (Barreda-Garcia et al., 2015; Barreda-Garcia et al., 2016; Motre et al., 2011). The optimal reaction mixture (50 μL) for HDA that could provide an amplification without any non-specificity, contained 25 μL sterile water, 5 μL 10x annealing buffer II, 2 μL MgSO_4_ (100 mM), 4 μL NaCl (500 mM), 3.5 μL IsoAmp® dNTP Solution, 1.5 μL primer mix comprising 5 μM forward and reverse primer each, 2 μL IsoAmp® enzyme mix, 2.5 μL EvaGreen (Thermo Fisher Scientific, USA), 2 μL ROX (Thermo Fisher Scientific), and 1 μL template. The optimal HDA reaction temperature was found to be between 67 and 68 °C.

### 2.2. Establishment of HDA over the TINY platform

After determining the optimal HDA reaction conditions for the detection of *IS6110* using ViiA7, HDA was examined for its compatibility with TINY. The HDA reaction mixture was prepared as described above and mixed well. Since TINY does not have a heated lid as in ViiA7, 30 μL of mineral oil (VWR International, USA) was overlaid on the reaction mixture to prevent evaporation. Then, TINY was operated under electric power and the results were interpreted by following the protocol as described previously (Snodgrass et al., 2018). HDA was performed in TINY using 2.5 × 10^9^ copies of pUCIDT-AMP vector carrying the *IS6110* in triplicates under the same temperature as mentioned above. For negative control, sterile water is used instead of template DNA in the reaction mixture. The reactions that showed a threshold time of less than 50 min were considered as positive. The threshold time is the time at which the relative fluorescence significantly increases above the baseline signal.

### 2.3. Limit of detection of HDA in TINY for the detection of IS6110

To determine the limit of detection of HDA, the pUCIDT-AMP vector carrying the *IS6110* was serially diluted 10-fold with sterile water from 2.5 × 10^9^ copies/μL to lower concentrations. Then, 1 μL of the diluted suspensions were subjected to HDA in TINY in triplicates and the respective threshold times were determined. The least concentration that had a threshold time of less than 50 min was determined as the limit of detection of HDA in TINY for *IS6110* detection.

### 2.4. Evaluation of HDA in TINY for mycobacterial strains

For evaluating the established HDA in TINY to detect the presence of *IS6110* in the Mtb pathogen, three *IS6110*-positive Mtb strains – H37Rv, CDC 1551, and Erdman wild-type and one *IS6110*-negative *Mycobacterium avium* strain were used. The genomic DNA was isolated from these strains using the procedure described before with slight modifications (van Helden et al., 2001). The isolated genomic DNA from the four strains was diluted with sterile water to a final concentration of 1 ng/μL and subjected to HDA in TINY in triplicate.

### 2.5. HDA in TINY for spiked oral swabs

To examine the utility of HDA in TINY for detecting Mtb directly from oral specimens, spiked oral swabs were used. Oral swabs were collected from a healthy volunteer and mixed with 1 mL sterile water. The pUCIDT-AMP vector carrying *IS6110* is mixed with the oral swab suspension at two different concentrations to reach a final concentration of 2.5 × 10^8^ copies/μL and 2.5 × 10^5^ copies/μL. Then, 1 μL of the oral swab suspension was subjected to HDA over the TINY platform. The unspiked orals swab suspension was used as a negative control and the results were evaluated. For repetition, different spiked oral swabs from the same person were used.

## 3. Results

### 3.1. Establishment of HDA in TINY for the qualitative detection of Mtb

Using 1 ng/μL pUCIDT-AMP vector carrying *IS6110* as positive control and sterile water as the negative control, HDA was performed in TINY in triplicates to examine its utility for the detection of Mtb. The threshold time for the detection of 2.5 × 10^9^ copies of *IS6110* template was found to be 23.25 ± 0.21 min. However, no amplification was observed in the negative control reaction with sterile water.

### 3.2. Limit of detection of HDA in TINY for the detection of IS6110

**Fig. 1.**
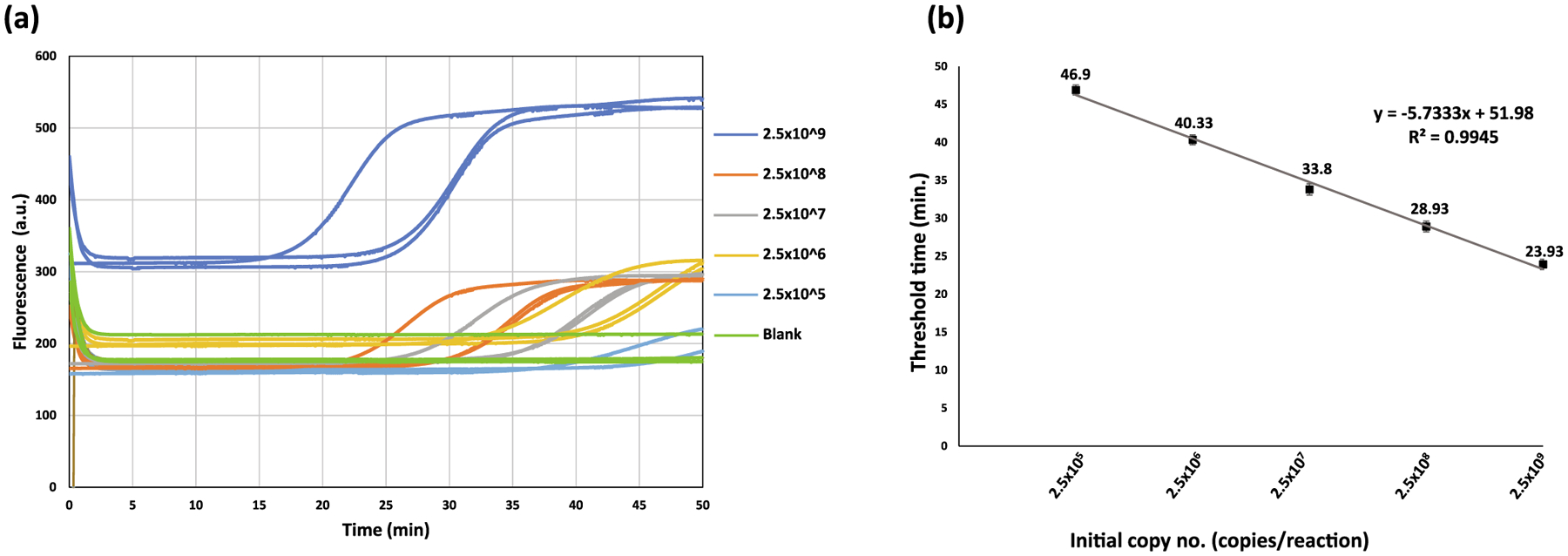
Limit of detection of HDA in TINY for *IS6110*. (a) The real-time fluorescence curves for the different copy numbers of IS6110 and the blank in triplicates. The triplicates are shown in the same color. (b) The linear plot between the threshold time and *IS6110* copy numbers. Error bars indicate the standard error of the mean.

The 10-fold serially diluted pUCIDT-AMP vector carrying the *IS6110* ranging from 2.5 × 10^9^ copies were subjected to HDA in TINY in triplicates. The lowest copy number of *IS6110* that could be detected by the HDA in TINY platform is 2.5 × 10^5^ copies/μl in 46. 45 ± 1.2 min (<50 min) threshold time. Fig. 1a shows the real-time fluorescence curves for the different copy numbers of *IS6110* and the blank. Furthermore, an increasing linear trend of threshold time was observed with the corresponding reduction in *IS6110* copy number (Fig. 1b).

### 3.3. HDA in TINY for mycobacterial strains

**Fig. 2.**
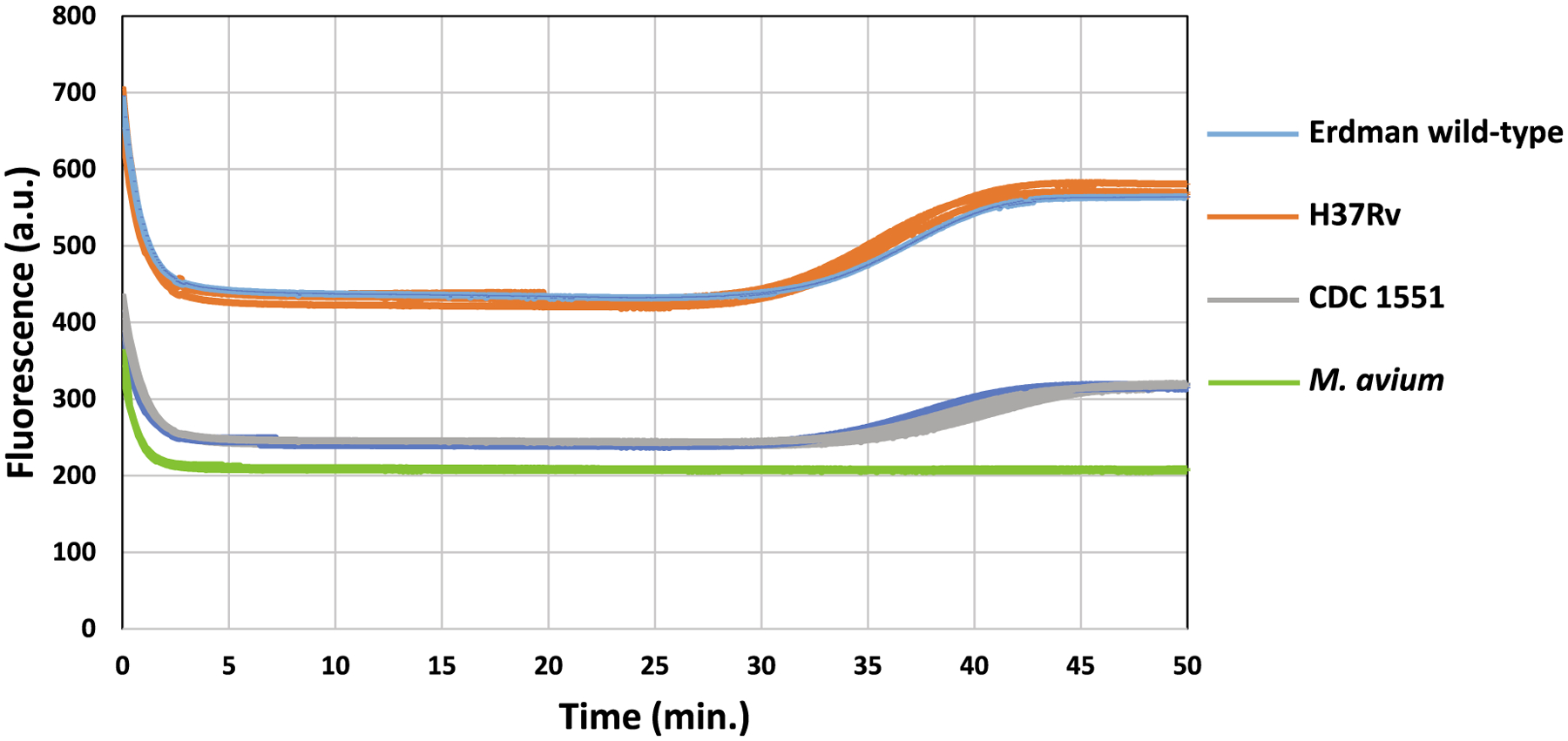
Examination of HDA in TINY for mycobacterial strains. The real-time fluorescence curves for the three *IS6110*-positive Mtb strains (H37Rv, CDC 1551, and Erdman wild-type) and the *IS6110*-negative *Mycobacterium avium* in triplicates. The triplicates are shown in the same color.

HDA was examined for the detection of *IS6110* in three *IS6110*-positive and one *IS6110*-negative mycobacterial strains. All three *IS6110*-positive strains showed positive for HDA with a threshold time ranging from ~28.36 ± 0.75 to 33.58 ± 0.79 min. The *IS6110*-negative strain (*M. avium*) showed no amplification in <50 min. In other words, the HDA in TINY platform can detect the Mtb pathogens without any non-specificity (Fig. 2).

### 3.4. HDA in TINY for spiked oral swabs

**Fig. 3.**
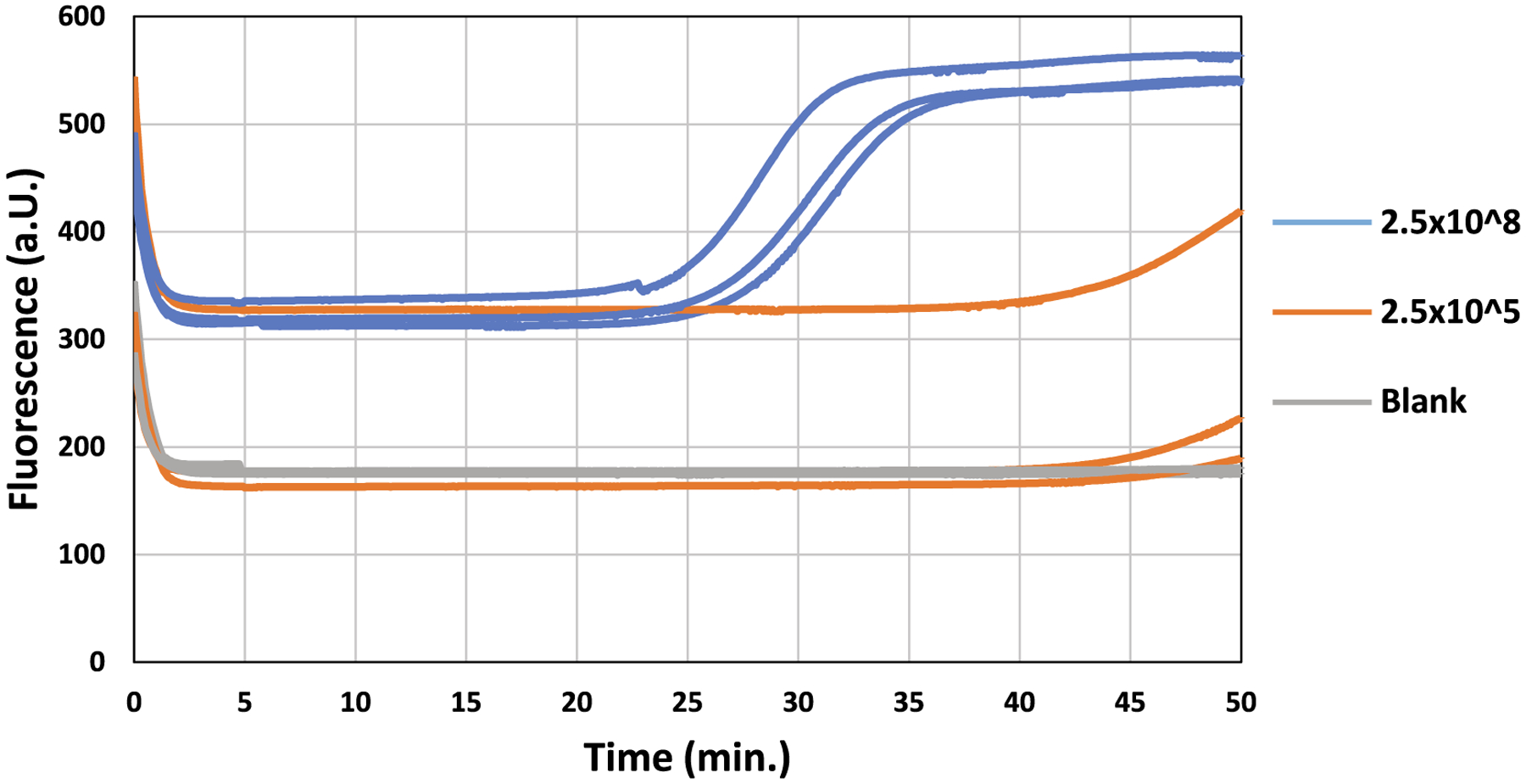
Evaluation of HDA in TINY for spiked oral swabs. The real-time fluorescence curves for the *IS6110*-spiked swabs (2.5 × 10^8^ and 2.5 × 10^5^ copies of *IS6110*) and the negative control spiked with sterile water. The triplicates are shown in the same color.

The diagnostic system was then examined for spiked oral swabs. The oral swabs spiked with 2.5 × 10^8^ and 2.5 × 10^5^ copies of pUCIDT-AMP vector carrying the *IS6110* showed amplification with a threshold time of <50 min. The oral swab spiked with sterile water (negative control) showed no amplification. Fig. 3 shows the compiled results of HDA in TINY for spiked oral swabs.

## 4. Discussion

**Fig. 4.**
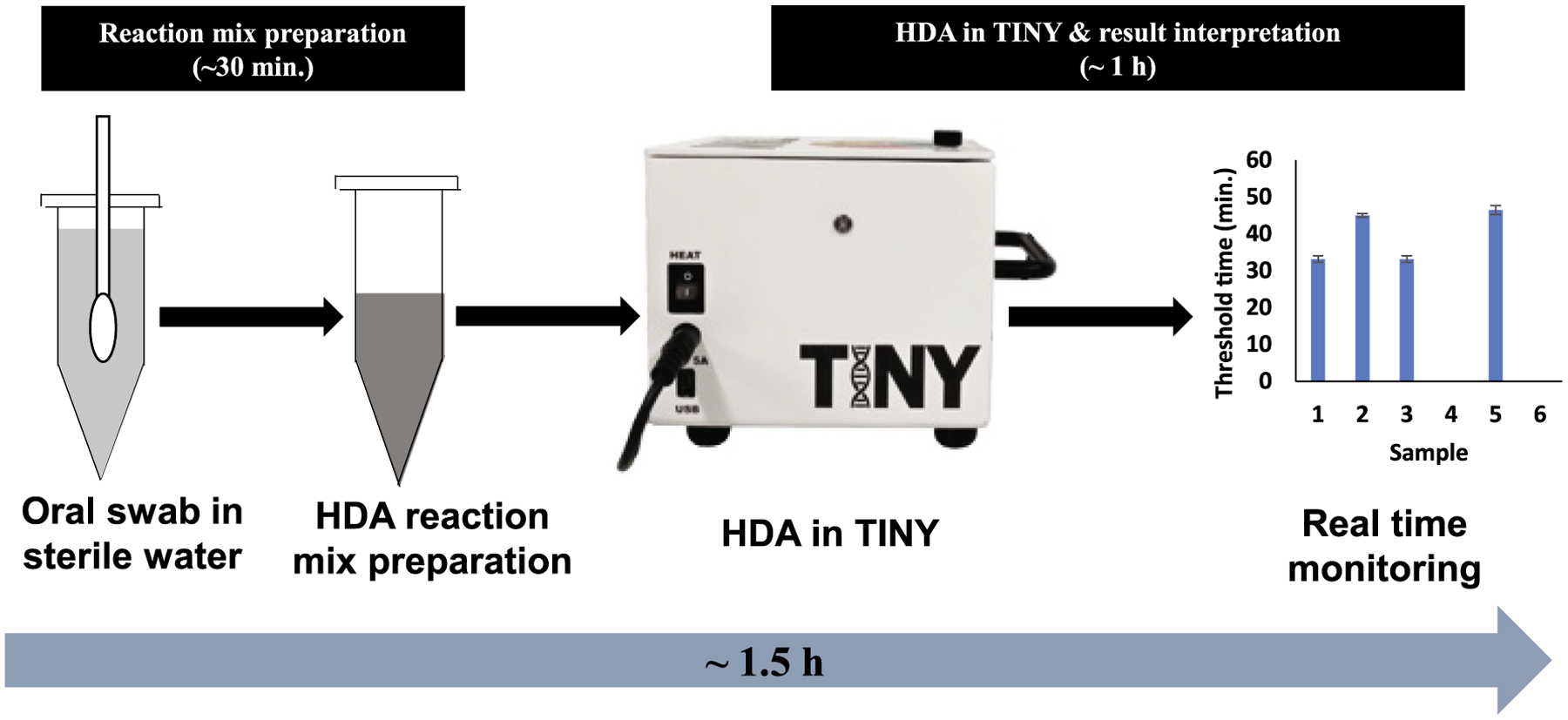
Schematic of the HDA in the TINY platform for TB diagnosis. The entire process from specimen preparation to result interpretation could be completed in <1.5 h.

In this manuscript, we report the utility of HDA over the TINY platform for the rapid diagnosis of Mtb directly from oral specimens. Our results showed that HDA in TINY could qualitatively detect the presence of Mtb by targeting the *IS6110* sequence in the Mtb genome. HDA in TINY could detect the presence of *IS6110* in Mtb strains without any non-specific identification of the negative control strain. Furthermore, it could detect the *IS6110* directly from the spiked oral swabs without any non-specific amplification demonstrating its utility for oral specimens. As it does not require any specialized sample preparation process for DNA isolation from oral swabs, it can be a more reliable diagnostic for TB. The specimen and HDA reaction mixture preparation took ~30 min. HDA reaction over the TINY platform and result interpretation took less than 1 h. Therefore, from specimen preparation to result interpretation, HDA in TINY requires less than 1.5 h to detect the presence of Mtb in oral swabs. The schematic for the detection of Mtb in oral swabs using HDA in TINY is described in Fig. 4.

TB is a major global health issue due to its high rate of morbidity and mortality. With the “End TB Strategy” by WHO, many health care programs are being implemented to control TB spread and infection. The timely and accurate diagnosis of TB is the cornerstone to initiate relevant treatment in the affected individuals as well as prevent the transmission of Mtb. The currently available diagnostics are often unable to be used for childhood TB as children have difficulty producing sputum. Further, a non-sputum-based diagnostic for TB particularly for children is also needed for the efficient control of TB.

Apart from the utility of HDA in TINY for oral swabs, our diagnostic platform can be applied in resource-limited clinical settings with limited infrastructure. TINY can be installed for routine clinical examination or can be used in the community setting. In addition to the electrical power supply, TINY also works on battery and solar power (Snodgrass et al., 2018). Moreover, it does not require any continuous maintenance or a major footprint which is required for other TB diagnostics like Xpert® MTB/RIF. The limit of detection of TINY for the detection of *IS6110* in Mtb is found to be 2.5 × 10^5^ copies/μl. The GeneXpert Assay has a limit of detection of 131 CFU/ml of clinical specimen (Marlowe et al., 2011). As this is a proof-of-concept study, performing the limit of detection with Mtb-positive clinical specimens is out of the scope due to funding, ethical approval, patient enrollment, and other related issues. It has been planned to perform a separate study for evaluating the limit of detection of TINY in terms of CFU/ml of the specimen and its validation such as sensitivity/specificity with real clinical specimens. As the primer set used in this study is already proved to have high selectivity for *IS6110* in Mtb by HDA (Barreda-Garcia et al., 2015; Barreda-Garcia et al., 2016; Motre et al., 2011), we have not performed a selectivity study here. The non-requirement of specimen preprocessing; the ability to use non-sputum samples; simplicity in operating the TINY system; and easier interpretation of results altogether positions the HDA in TINY as an efficient diagnostic platform for TB.

## Supplementary Material

1

## Abbreviations:

TB: active tuberculosis disease
Mtb: Mycobacterium tuberculosis
TINY: Tiny Isothermal Nucleic acid quantification sYstem
HDA: helicase-dependent amplification
WHO: World Health Organization
LMICs: low- and middle-income countries
POC: point-of-care
