# Supplementary material for: An isothermal amplification-based point-of-care diagnostic platform for the detection of Mycobacterium tuberculosis: A proof-of-concept study
Source: Curr Res Biotechnol. Author manuscript; Available in PMC 2021 Jul 23. (PMC8301208; doi:10.1016/j.crbiot.2021.05.004)
Supplement: 1 [file NIHMS1723624-supplement-1.docx]

**SUPPLEMENTARY MATERIAL**

**Table S1**

List of primer sets evaluated for their efficient detection of *IS6110* by HDA.

| **Primer name** | **Forward (F)/ Reverse (R)** | **Sequence (5’-3’)** | **Amplicon size** | **References** |
| --- | --- | --- | --- | --- |
| IS6110-1 | F | acaagaaggcgtactcgacctgaaagacgttat | 81 | This study |
|  | R | tcgctgaaccggatcgatgtgtactgagat |  |  |
| IS6110-2 | F | atcaacggcctatacaagaccgagctgatcaaa | 124 | This study |
|  | R | agtactggtagaggcggcgatggttgaa |  |  |
| IS6110-3 | F | gcggtcggaagctcctatgacaatgcacta | 80 | This study |
|  | R | ttgccgggtttgatcagctcggtctt |  |  |
| IS6110-4 | F | cgatggcgaactcaaggagcacatca | 94 | This study |
|  | R | ctcacggttcagggttagccacacttt |  |  |
| IS6110-5 | F | tgtttacggtgcccgcaaagtgtggctaa | 88 | This study |
|  | R | tttggtcatcagccgttcgacggtgcatct |  |  |
| IS6110-6 | F | atctcagtacacatcgatccggttcagc | 111 | This study |
|  | R | ttgatcgtctcggctagtgcattgtcata |  |  |
| IS6110-7 | F | atggcgaactcaaggagcacatca | 89 | This study |
|  | R | acggttcagggttagccacacttt |  |  |
| IS6110-8 | F | cccatcgacctactacgaccacatcaac | 128 | This study |
|  | R | acactttgcgggcaccgtaaacac |  |  |
| IS6110-9 | F | ctcagtacacatcgatccggttcagcga | 112 | This study |
|  | R | ccgttgatcgtctcggctagtgcattgt |  |  |
| IS6110-10 | F | caacaagaaggcgtactcgacctga | 84 | (Barreda-Garcia et al., 2016) |
|  | R | ctcgctgaaccggatcgatgtgtact |  |  |

**References**

Barreda-Garcia, S., Miranda-Castro, R., de-Los-Santos-Alvarez, N., Miranda-Ordieres, A.J., Lobo-Castanon, M.J., 2016. Comparison of isothermal helicase-dependent amplification and PCR for the detection of Mycobacterium tuberculosis by an electrochemical genomagnetic assay. Anal Bioanal Chem. 408, 8603-8610. doi: 10.1007/s00216-016-9514-z
